# Association of plasma macrophage colony-stimulating factor with cardiovascular morbidity and all-cause mortality in chronic hemodialysis patients

**DOI:** 10.1186/s12882-019-1510-z

**Published:** 2019-08-16

**Authors:** Xuan Deng, Qian Yang, Yuxi Wang, Yi Yang, Guangchang Pei, Han Zhu, Jianliang Wu, Meng Wang, Zhi Zhao, Huzi Xu, Cheng Zhou, Yi Guo, Ying Yao, Zhiguo Zhang, Wenhui Liao, Rui Zeng

**Affiliations:** 1 Department of Nephrology, Tongji Hospital Affiliated with Tongji Medical College, Huazhong University of Science and Technology, Wuhan, Hubei 430030 People’s Republic of China; 2 Department of Geriatrics, Tongji Hospital Affiliated with Tongji Medical College, Huazhong University of Science and Technology, Wuhan, Hubei 430030 People’s Republic of China; 3 School of Medicine and Health Management, Tongji Medical College, Huazhong University of Science and Technology, Wuhan, Hubei 430030 People’s Republic of China

**Keywords:** M-CSF, CVD events, All-cause mortality, Hemodialysis

## Abstract

**Background:**

Cardiovascular disease (CVD) events are the main cause of death in long-term hemodialysis (HD) patients. Macrophage colony- stimulating factor (M-CSF) is actively involved in the formation of atherosclerosis and causes plaque instability, thrombosis and the development of acute coronary syndromes. However, little information is available on the role of M-CSF in HD patients. We aimed to investigate the association between plasma M-CSF levels and CVD events as well as all-cause mortality in patients undergoing long-term HD.

**Methods:**

Fifty two HD patients and 8 healthy controls were recruited in this study. HD patients were followed up from September 2014 to May 2017. The primary end point was CVD event, the secondary outcome was death from any cause. Patients were divided into two groups with low and high M-CSF levels based on the optimal cut-off value determined by the ROC curve. Cox regression analyses were used to assess the predictive value of plasma M-CSF for CVD events and all-cause mortality in HD patients. We tested the levels of plasma M-CSF and other inflammatory cytokines in surviving HD patients using ELISA or CBA kit.

**Results:**

The average plasma level of M-CSF in 52 patients was approximately twice that of healthy controls (992.4 vs. 427.2 pg/mL; *p* <  0.05). During 32 months of follow-up, 26 patients (50.0%) had at least one CVD event and 8 patients (15.4%) died. The mean plasma M-CSF concentration increased in survivors after follow-up compared to that detected at baseline (1277.8 ± 693.3 vs. 997.2 ± 417.4 pg/mL; *p* <  0.05). Multivariate Cox regression analysis showed that plasma M-CSF is an independent risk factor for CVD events in HD patients (*p* <  0.05). In the Cox regression model after adjusting for gender and age, high M-CSF levels were related to an increased risk of all-cause death (*p* <  0.05). We also found that M-CSF levels were positively correlated with IL-6 and IL-18 levels (both *p* < 0.05), which are the major pathogentic cytokines that contribute to HD-related CVD events.

**Conclusion:**

M-CSF is a prognostic factor for CVD events and all-cause mortality in HD patients.

## Background

Cardiovascular disease (CVD) is the leading cause of mortality in patients with chronic hemodialysis (HD) [1, 2]. The underlying causes include inflammation, atherosclerosis, volume overload and malnutrition [3]. Cardiovascular death in dialysis patients is reckoned to be 10 to 20 times higher than that in the general population [4]. Therefore, the assessment of the risk of cardiovascular events is the principal goal to improve the prognosis of patients with maintenance hemodialysis.

Macrophage colony-stimulating factor (M-CSF), also known as CSF-1, is the primary regulator of mononuclear phagocytes survival, proliferation, differentiation [5, 6] and stimulates inflammation that leads to macrophage-mediated destruction [7]. Mononuclear cells from peripheral blood infiltrated into atherosclerotic plaques, respond to M-CSF, differentiate into activated macrophages and play an important role in the development of atherosclerosis [8]. In addition, M-CSF is the main trigger factor for the production of tissue factor inducing plaque thrombosis and instability, and in turn causes acute coronary syndrome [9]. Several studies showed the upregulation of serum M-CSF in patients with angina pectoris [10, 11] and acute myocardial infarction [10, 12]. Hohensinner PJ reported that M-CSF can predict the adverse outcome of heart failure [13]. Being consistent with that, Rallidis LS’ research showed that high M-CSF is an independent predictor of adverse events in hospitalized patients [14] and the high level of M-CSF at the end of 6-week follow-up is a strongly predictor of long-term adverse outcome in patients with serious unstable angina [15]. However, there is little information about the long-term prognostic value of M-CSF in CVD events and deaths in patients undergoing long-term hemodialysis.

A five-year follow-up study found that M-CSF is significantly associated with annual decline in estimated glomerular filtration rate (eGFR) [16]. The decrease of eGFR is closely related to the risk of end-stage renal disease, CVD events and mortality [17–20]. Therefore, our study is to evaluate the prognostic value of plasma M-CSF level in cardiovascular disease and death in patients undergoing maintenance hemodialysis.

## Methods

### Design and patients

This was a single-center, observational, prospective study that recruited 52 patients undergoing maintenance hemodialysis. The study was conducted from September 2014 to May 2017. All patients underwent a baseline visit at enrollment and a follow-up visit at 32 months. Patients with stable clinical data and dialysis for at least three months were included. Exclusion criteria included diagnosed with cardiac disease, malignancy or other active neoplasia, active inflammation, immunosuppressive therapy, chemotherapy or radiation therapy in the past three months, a scheduled or recently failed organ transplant, a scheduled adjustment of dialysis mode or a plan to other hospitals for treatment. All patients were subjected to conventional hemodialysis procedures, undergoing standard bicarbonate hemodialysis, thrice-weekly for 4 h each time in-center hemodialysis. Both patients and volunteers were notified about the purpose of the study, consented to participate in the study and signed informed consent. We recorded the clinical and demographic data, the cardiovascular events and deaths of hemodialysis patients during the 32-month follow-up. At the end of the follow-up, we measured the levels of plasma M-CSF and inflammatory cytokines in surviving hemodialysis patients.

### Data collection and follow-up

Demographics, clinical and biochemical data of patients were collected through patient interviews, medical records and the reports of the doctors, including age, sex, hemoglobin, serum creatinine, serum urea nitrogen, serum uric acid, total cholesterol, serum potassium, serum calcium, serum phosphate, parathyroid hormone. Patients were visited until death, and adverse events during the long-term follow-up (CVD events and all-cause mortality) were recorded through medical records, face to face or telephone interview. CVD was defined as documented arrhythmia, acute coronary syndrome, myocardial infarction, congestive heart failure, cerebrovascular disease, or peripheral vascular disease. The primary end point was CVD event, the secondary outcome was death from any cause. All patients were visited at least three times.

### Measurement of plasma M-CSF

Blood were taken before the first dialysis session of the week and before administration of medications from patients. As a control, we obtained blood from healthy volunteers with negative proteinuria and normal creatinine. All blood samples were collected within one week. It was immediately centrifuged at 3000 rpm for 10 min within one hour of obtaining the sample. Plasma was frozen in aliquots and stored at − 80 °C to avoid repeated freezing and thawing. Plasma M-CSF levels were measured using a commercially available trusted ELISA kit (R&D Systems®, Catalog # PDMC00B). The minimum detectable dose for M-CSF is ranged from 1.74 to 47.3 pg/mL.

### Measurement of plasma inflammatory cytokines

The plasma levels of IL-1β, IL-6, IL-8, IL-18, IL-23, TNF-α were detected by cytometric bead array, according to the manufacturer’s protocol (Biolegend). Data were obtained by flow cytometry and analyzed by FCS Express 6 Plus provided by the company. The value below the detection limit on each well was set to 0.

### Statistical analysis

Statistical analysis and data presentation were performed using SPSS 19.0 (IBM Corp, Chicago IL, USA) and GraphPad Prism 6. Continuous variables were compared by the independent-samples t-test, if not, the Mann–Whitney test was used. Categorical variables were analyzed by the Chi-square statistics. Data are presented as mean ± SD, median (interquartile range) or percentage (%).The receiver operating characteristic (ROC) curve was performed to calculate the threshold of M-CSF levels for CVD events and all-cause mortality respectively. The value with the maximum Youden index was selected as the best cut-off point. The event-free survival rates of CVD events and all-cause mortality were examined by Kaplan-Meier method, and compared by log-rank test. To assess the relationship between M-CSF elevation and cardiovascular morbidity or all-cause mortality, we performed uni- and multi-variate COX regression analysis. The results presented as odds ratio (OR) and 95% confidence interval (CI). All statistical analyzes were two-sided, significance was set at *p* < 0.05.

## Results

### Plasma levels of M-CSF

**Fig. 1:**
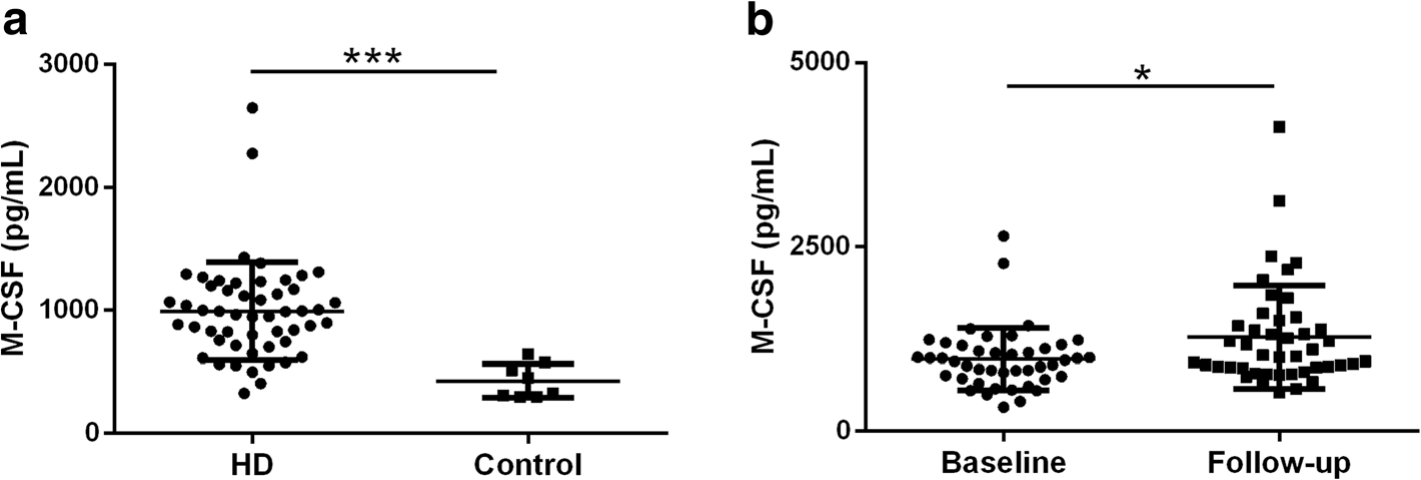
**a** Comparison of plasma M-CSF levels in healthy controls and HD patients at baseline, *p* = 0.0002. **b** M-CSF levels at baseline and the end of follow-up in 44 HD patients, *p* = 0.017. * denotes *p* < 0.05, ** denotes *p* < 0.01, *** denotes *p* < 0.001. M-CSF, macrophage colony-stimulating factor

The average plasma level of M-CSF in 52 patients was 992.4 pg/mL (range 325.58 ~ 2647.08), which was approximately twice that of normal donors (427.2 pg/mL, range 296.56 ~ 644.82) (Fig. 1a). At the end of the study period, there were 44 patients survived. We collected blood samples from these surviving patients and measured the concentration of M-CSF. We found that the mean level of plasma M-CSF in this test (1277.78 ± 693.33 pg/mL) was significantly higher than that (997.24 ± 417.39 pg/mL) detected 32 months ago (Fig. 1b).

### Baseline characteristics of study population

Among the 52 patients with HD, 29 were male and 23 were female, with an average age of 54.8 years. Of the 8 healthy volunteers, 4 were males and 4 were females, with a mean age of 49.9 years. There was no statistically significant difference in age and sex between the two groups. However, compared to the control subjects, the HD group had higher serum creatinine, serum urea nitrogen, serum uric acid. The levels of hemoglobin and total cholesterol were significantly reduced in HD patients as compared to the control group. The above and other parameters were presented in Table 1. After the 32-month follow-up, the average plasma level of M-CSF in 44 survivors increased significantly. The difference between 2014 and 2017 was evaluated as a ΔM-CSF, and patients were divided into two groups according to median value of ΔM-CSF, ΔM-CSF-high group and ΔM-CSF-low group. To assess the effect of M-CSF increase on patients, we compared the characteristics of the two groups at the end of follow-up. Groups were similar regarding age, gender, hemodialysis duration, prevalence of diabetes, serum uric acid, serum potassium, serum calcium, serum phosphate, alkaline phosphatase, systolic BP, diastolic BP, mean BP. However, patients with rapid M-CSF growth suffered higher serum creatinine, blood urea, intact parathyroid hormones, and lower hemoglobin (Table 2).

**Table 1:** Baseline characteristics of HD patients and healthy controls

Variables	HD patients	healthy controls	*p* value
Number	52	8	
Male (%)	55.77%	50%	> 0.05
Age (Y)	54.75 ± 17.86	49.88 ± 7.28	> 0.05
Hb (g/L)	104.04 ± 17.39	146.13 ± 15.09	< 0.0001
Serum creatinine (μmol/L)	931.13 ± 270.55	72.50 ± 14.83	< 0.0001
Serum urea nitrogen (mmol/L)	26.58 (21.38,31.37)	4.50 (4.43,4.93)	< 0.0001
Serum uric acid (μmol/L)	441.19 ± 122.88	338.99 ± 60.04	< 0.05
Total cholesterol (mmol/L)	3.68 ± 0.79	4.99 ± 0.62	< 0.0001
K (mmol/L)	5.10 ± 0.92		
Ca (mmol/L)	2.21 ± 0.27		
P (mmol/L)	1.98 ± 0.62		
IPTH (ng/L)	305.8 (149.7471.5)		
Predialysis Systolic BP (mmHg)	143.62 ± 19.81		
Predialysis Diastolic BP (mmHg)	83.94 ± 13.60		
Predialysis Mean BP (mmHg)	103.83 ± 13.59		

Data are presented as percentage (%), mean ± SD or median (interquartile range)

*HD* hemodialysis, *Hb* hemoglobin, *K* kalium, *Ca* calcium, *P* phosphorus, *IPTH* intact parathyroid hormone, *BP* blood pressure

**Table 2:** Clinical characteristics of the ΔM-CSF-high and the ΔM-CSF-low groups at the end of follow-up

Variables	△M-CSF-low group	△M-CSF-high group	*p* value
Number	22	22	
Male (%)	50%	68%	0.220
Age (Y)	57.14 ± 15.51	55.64 ± 19.24	0.777
Hemodialysis duration (months)	93.97 ± 43.17	82.59 ± 36.27	0.349
Diabetes (%)	13.6%	22.7%	0.434
Predialysis Hb (g/L)	112.18 ± 11.04	101.00 ± 18.97	0.021
Predialysis serum creatinine (μmol/L)	970.79 ± 209.40	1111.50 ± 242.59	0.049
Predialysis serum urea nitrogen (mmol/L)	25.67 ± 5.00	30.08 ± 6.69	0.020
Predialysis serum uric acid (μmol/L)	466.00 (436.50,512.00)	465.00 (406.80,554.00)	0.966
Predialysis K (mmol/L)	5.10 ± 0.80	4.96 ± 0.96	0.582
Predialysis Ca (mmol/L)	2.46 (2.12–2.53)	2.30 (2.16–2.48)	0.425
Predialysis P (mmol/L)	2.07 ± 0.55	2.18 ± 0.62	0.318
Predialysis IPTH (ng/L)	384.40 (153.40,688.00)	536.30 (243.68,1595.00)	0.049
Predialysis Systolic BP (mmHg)	147.00 (123.00,162.50)	140.00 (118.30,160.00)	0.527
Predialysis Diastolic BP (mmHg)	82.10 ± 13.51	80.73 ± 13.56	0.742
Predialysis Mean BP (mmHg)	102.06 ± 14.70	99.76 ± 14.46	0.607

*M-CSF* macrophage colony- stimulating factor, *Hb* hemoglobin, *IPTH* intact parathyroid hormone, *BP* blood pressure

### Associations of M-CSF levels with CVD events

**Fig. 2:**
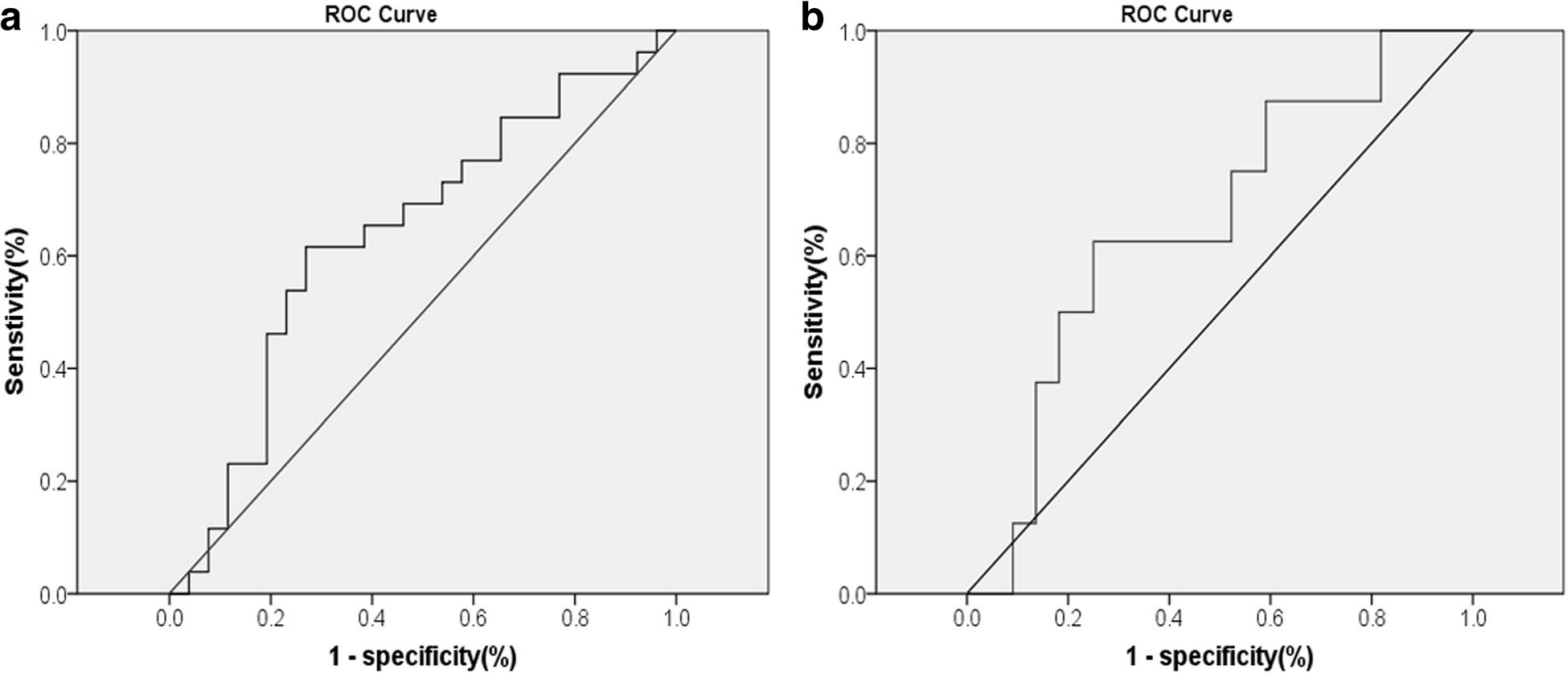
ROC curve was used to calculated the optimal cut-off value of M-CSF predicting (**a**) CVD events (AUC = 0.639; 95%CI 0.485~0.793; sensitivity = 61.5%, specificity = 73.1%) and (**b**) all-cause mortality (AUC = 0.659; 95%CI 0.462~0.856; sensitivity = 62.5%, specificity = 75%), respectively. ROC, receiver operating characteristic; AUC, area under the ROC curve; CI, confidence interval; CVD, cardiovascular disease

**Fig. 3:**
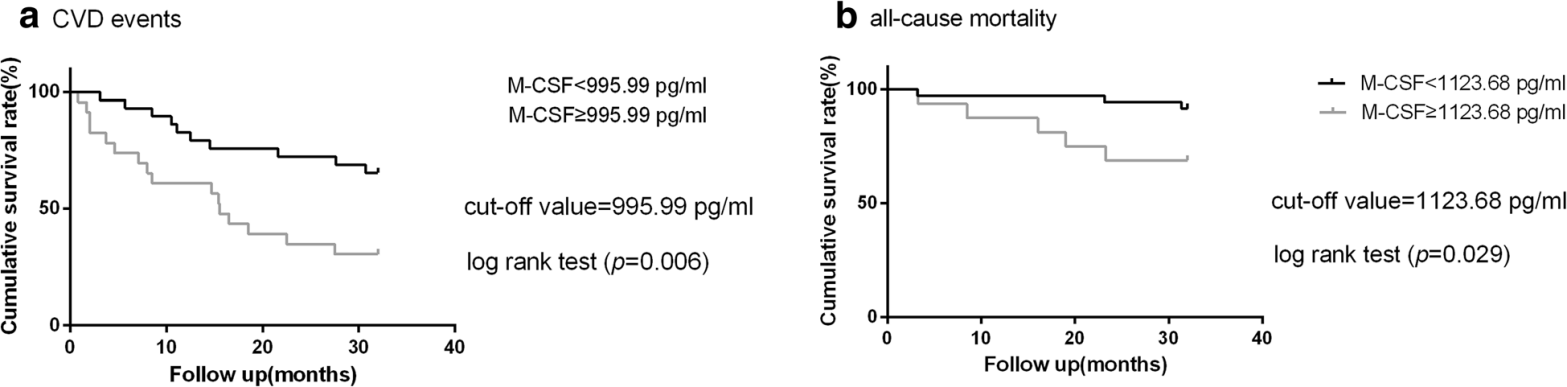
Kaplan-Meier survival curves for **a** CVD events and **b** all-cause mortality

During the 32-month follow-up period, 26 patients (50%) suffered at least one CVD event, and another 26 patients had no history of CVD events. Optimal cut-off value (995.99 pg/mL) of the M-CSF level for CVD prediction was determined using receiver operating characteristic (ROC) curve (Fig. 2a). Patients were split into two groups, with low and high M-CSF levels, according to the best cut-off value. The Kaplan–Meier curve (Fig. 3a) confirmed that patients with high M-CSF had significantly higher incidence rates of CVD events (log-rank test, *p* < 0.05). Several vital factors, including plasma M-CSF levels, were analyzed by univariate cox analysis (Table 3). Of the 8 vital variables, factors including age, systolic BP and levels of M-CSF were identified as significant predictors (HR = 1.04 [1.01~1.07], 1.02 [1.00~1.04], 2.89 [1.30~6.40], respectively; all *p* < 0.05). Then we established four different cox regression models, adjusted for some important confounding risk factors in each model, and found that M-CSF is an independent risk factor for CVD events in HD patients (all *p* < 0.05).

**Table 3:** Cox regression analyses of the association of the important variables with cardiovascular disease morbidity

Variables	HR	95% CI	*p* value
Univariate analyses (*n* = 52)			
Sex(M/F)^a^	1.084	0.497–2.364	0.840
Age (Y)	1.039	1.013–1.065	0.003
Hb (g/L)	1.016	0.994–1.040	0.159
Predialysis Serum creatinine (μmol/L)	0.999	0.997–1.000	0.117
Predialysis Systolic BP (mmHg)	1.023	1.002–1.044	0.035
Predialysis Diastolic BP (mmHg)	0.982	0.952–1.013	0.257
Predialysis Mean BP (mmHg)	1.005	0.975–1.036	0.754
M-CSF ^b^	2.889	1.305–6.398	0.009
Multivariate analyses(*n* = 52)			
M-CSF (adjusted for sex, age, systolic BP)	4.504	1.851–10.959	0.001
M-CSF (adjusted for sex, age, HB)	4.728	1.985–11.264	< 0.001
M-CSF (adjusted for sex, age, serum creatinine)	4.932	2.013–12.082	< 0.001
M-CSF (adjusted for sex, age, Diastolic BP)	4.506	1.894–10.721	0.001

^a^ Female has been codified as 0 and male as 1

^b^ M-CSF concentration < 995.99 pg/mL has been codified as 0 and ≥ 995.99 pg/mL as 1

*HR* Hazard ratio, *CI* confidence interval, *BP* blood pressure

### Association of M-CSF levels with death

After an average of 29.9 months follow-up (3.2–32.0 months), 8 out of 52 patients (15.4%) met the secondary endpoint of any cause. Receiver operating characteristic (ROC) curve (Fig. 2b) was carried out to calculate the cut-off value of the M-CSF level (1123.68 pg/mL). Patients were divided into two groups according to the M-CSF level threshold, one with high M-CSF and one with low M-CSF. Five patients died in the high M-CSF level group and three patients died in the low M-CSF level group. The Kaplan–Meier curve (Fig. 3b) showed that time to death of any cause was significantly reduced with high M-CSF group (log-rank test, *p < 0.05*). The univariate Cox regression confirmed that the group with high M-CSF had significantly higher unadjusted HR for all-cause mortality compared with the other group (unadjusted HR = 4.31 [1.03~18.08]; *p < 0.05*). Using the multivariable Cox proportional hazards model, which adjusted vital factors of age and gender, we discovered that all-cause mortality was still significantly higher in the group with high M-CSF (adjusted HR = 4.30 [1.01~18.23]; *p < 0.05*), the result was presented in Table 4. The results indicated that high M-CSF levels were associated with increased risk of all-cause mortality in patients undergoing maintenance hemodialysis.

**Table 4:** Uni-and multivariate Cox regression analysis of the association of M-CSF levels with all-cause mortality

Variables	HR	95% CI	*p* value
Univariate analyses (*n* = 52)			
M-CSF	4.311	1.028–18.082	0.046
Multivariate analyses (*n* = 52)			
M-CSF (adjusted for sex,age)	4.298	1.013–18.234	0.048

M-CSF concentration < 1123.68 pg/mL has been codified as 0 and ≥ 1123.68 pg/mL as 1

*M-CSF* macrophage colony- stimulating factor, *HR* Hazard ratio, *CI* confidence interval

### Relationship between plasma concentration of M-CSF and other cytokines

**Fig. 4:**
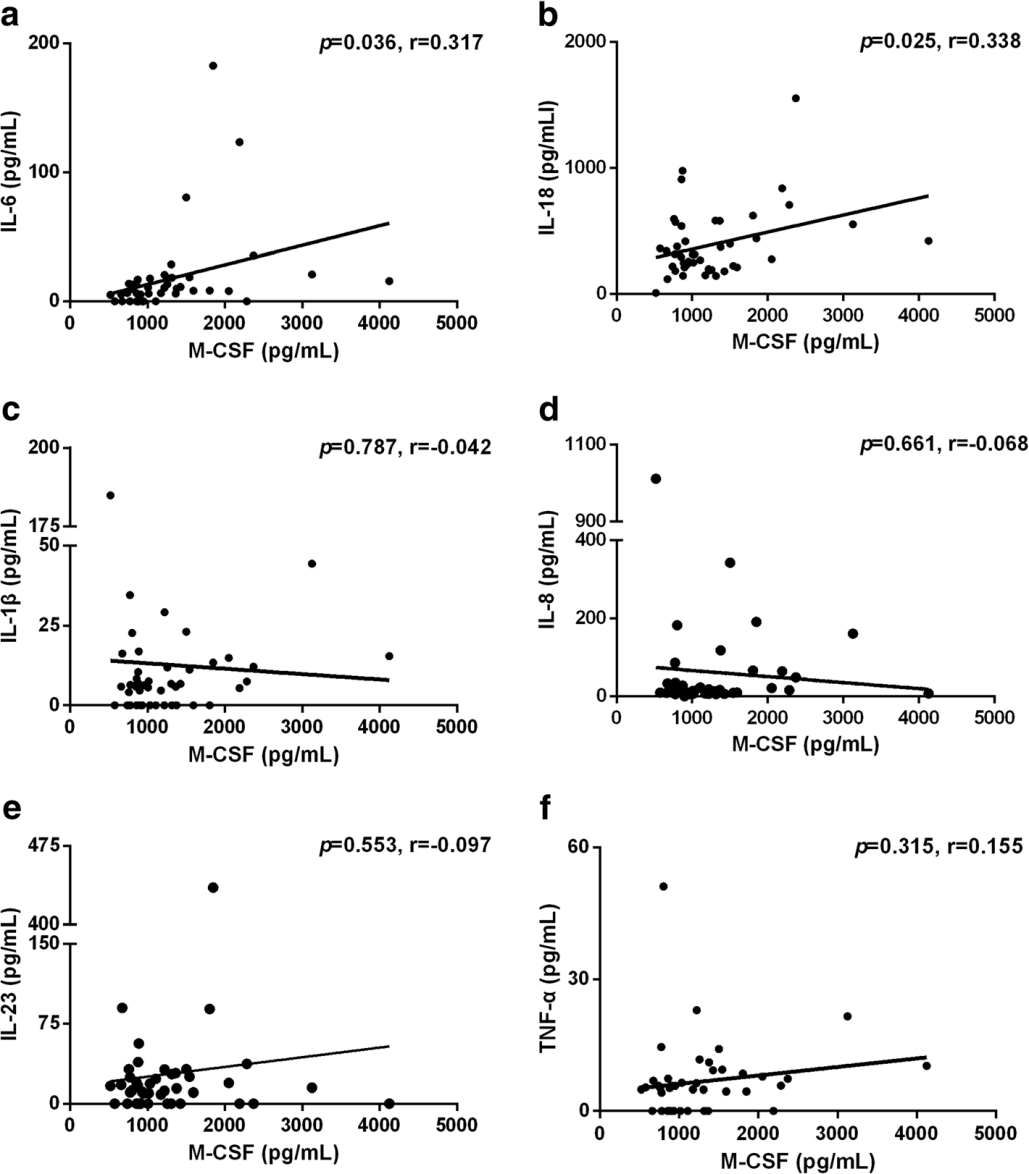
The relationship between plasma levels of M-CSF and (**a**) IL-6, (**b**) IL-18, (**c**) IL-1β, (**d**) IL-8, (**e**) IL-23, (**f**) TNF-α.

Linear regression analysis showed that M-CSF concentration was positively correlated with IL-6 (Fig. 4a) and IL-18 (Fig. 4b) concentration in 44 subjects, respectively (r = 0.32, *p* = 0.036; r = 0.34, *p* = 0.025). No correlation was apparent between M-CSF concentration and IL-1β (Fig. 4c), IL-8 (Fig. 4d), IL-23 (Fig. 4e) or TNF-α (Fig. 4f) concentration.

## Discussion

In the present observational analysis with 32 months of follow-up, plasma M-CSF levels were significantly predictive of cardiovascular events, as well as all-cause mortality. We found that plasma levels of M-CSF elevated in hemodialysis patients compared with healthy volunteers, which was consistent with other reports [21, 22].

It was reported that there was no difference in M-CSF levels before and after HD, which excluded the impact of the dialysis procedure on M-CSF production. So, what is the reason for the significant increase of plasma M-CSF levels in HD patients? We suppose the reasons are as follows: first, hemodialysis leads to endothelial dysfunction [23], which leads to increased secretion of M-CSF in the circulation. Second, M-CSF may accumulate in the blood because of decreased renal excretion [24]. Third, it was reported that compared with the control group, the expression of M-CSF gene in monocytes is significantly increased in HD patients. It is speculated that M-CSF gene expression should be downregulated in HD patients owing to their high plasma M-CSF levels, but this downregulation seems to be disrupted [25].

It had been reported that there was a negative correlation between plasma M-CSF level and left ventricular ejection fraction in patients undergoing long-term hemodialysis, and the increase of M-CSF exist before the development of left ventricular dysfunction [21]. However, the underlining mechanism of M-CSF on cardiovascular disease is still unclear, which may be a comprehensive result of multiple interactions. M-CSF is mainly released from endothelial cells, causes monocyte and macrophage activation [26], and mediates monocyte-induced death of smooth muscle cells, increased expression of metalloproteinases, and thus weakens the coronary plaque [27, 28]. M-CSF induces macrophages to express both scavenger receptors and CD36, thereby accelerating macrophage uptake of oxidized low density lipoproteins and formation of foam cells [25, 29, 30]. M-CSF stimulates monocyte, macrophages, vascular endothelial cells to release tissue factor (TF), leading to thrombosis [31]. Those data suggest that M-CSF directly contribute to the development of atherosclerosis.

M-CSF has also been identified to accelerate and multiply chronic inflammatory response. For example, it can promote the release of monocyte chemoattractant protein 1 (MCP-1) and IL-6, thereby further inducing TF expression [32]. MCP-1 is an common recognized independent prognostic indicator in acute and chronic phase of acute coronary syndrome [33] and proved to be a predictor of all-cause mortality in patients with heart failure [13]. IL-6 drives C-reactive protein (CRP) production [34], which has a direct proinflammatory impact on endothelial cells [35], and mediates the absorption of low density lipoprotein by macrophages [36] and induces apoptosis of coronary artery smooth muscle cells [37]. In this study, we found that M-CSF is positively correlated with IL-6 and IL-18, suggesting M-CSF induced activation of IL-6 is related to M-CSF associated cardiovascular disease in patients undergoing maintenance hemodialysis. IL-18, known as IFN-γ inducing factor, is in charge of the up-regulation of other inflammatory cytokines and adhesion molecules and contributes to the weakening of the extracellular matrix of plaque, which is the primary cause of coronary thrombosis [38]. In addition, animal models have demonstrated the beneficial effects of restraining IL-18 on plaque composition and progression [39]. The positive correlation between M-CSF and IL-18 suggests that they may have a synergistic effect on atherosclerosis in hemodialysis.

In addition, previous study showed that M-CSF inhibits the growth of erythroid progenitor cells in patients with renal failure who were undergoing hemodialysis, probably by reducing their sensitivity to erythropoietin [40]. Our finding was consistent with this conclusion. Anemia promotes heart ischemia by endothelial dysfunction-related atherosclerosis and reducing oxygen transport [41, 42]. Through these actions, M-CSF may also contribute to cardiovascular events and death in patients undergoing maintenance hemodialysis.

## Conclusion

In our prospective study of patients with HD, M-CSF was a powerful predictor of adverse events during 32 months follow-up. It was indentified as a particularly useful prognostic marker for concerning risk of time-course events in patients with HD. In the future, therapeutic approaches to target M-CSF and it associated cytokines are likely to alleviate an inflammatory status, reduce the occurrence of adverse events and benefit hemodialysis patients. Our study had some limitations. First of all, due to the limited scale of research, relatively few patients limited the statistical power of the results. Secondly, these patients may have atherosclerosis before enrollment due to lack of ultrasound findings, which already have a higher risk of cardiovascular disease. Finally, the subjects included in our study were all Asian and the findings may not apply to other races. A prospective confirmation of the findings and an analysis of a larger, more-diverse population are necessary.

## Data Availability

The datasets used and analysed during the current study are available from the corresponding author on reasonable request.

## Abbreviations

AUC: Area under the ROC curve
BP: Blood pressure
Ca: Calcium
CI: Confidence interval
CVD: Cardiovascular disease
Hb: Hemoglobin
HD: Hemodialysis
HR: Hazard ratio
IPTH: Intact parathyroid hormone
K: Kalium
M-CSF: Macrophage colony-stimulating factor
P: Phosphorus
ROC: Receiver operating characteristic
